# Heat Treatment of NiTi Alloys Fabricated Using Laser Powder Bed Fusion (LPBF) from Elementally Blended Powders

**DOI:** 10.3390/ma15093304

**Published:** 2022-05-05

**Authors:** Agnieszka Chmielewska, Bartłomiej Wysocki, Piotr Kwaśniak, Mirosław Jakub Kruszewski, Bartosz Michalski, Aleksandra Zielińska, Bogusława Adamczyk-Cieślak, Agnieszka Krawczyńska, Joseph Buhagiar, Wojciech Święszkowski

**Affiliations:** 1 Faculty of Material Science and Engineering, Warsaw University of Technology, Woloska 141 Str., 02-507 Warsaw, Poland; miroslaw.kruszewski@pw.edu.pl (M.J.K.); bartosz.michalski@pw.edu.pl (B.M.); ozielinska5@gmail.com (A.Z.); boguslawa.cieslak@pw.edu.pl (B.A.-C.); agnieszka.krawczynska@pw.edu.pl (A.K.); 2 Centre of Digital Science and Technology, Cardinal Stefan Wyszynski University in Warsaw, Woycickiego 1/3, 01-938 Warsaw, Poland; b.wysocki@uksw.edu.pl (B.W.); p.kwasniak@uksw.edu.pl (P.K.); 3 Department of Metallurgy and Materials Engineering, University of Malta, MSD 2080 Msida, Malta; joseph.p.buhagiar@um.edu.mt

**Keywords:** elemental powders, heat treatment, in situ alloying, laser powder bed fusion, nickel-titanium, pre-mixed powders

## Abstract

The use of elemental metallic powders and in situ alloying in additive manufacturing (AM) is of industrial relevance as it offers the required flexibility to tailor the batch powder composition. This solution has been applied to the AM manufacturing of nickel-titanium (NiTi) shape memory alloy components. In this work, we show that laser powder bed fusion (LPBF) can be used to create a Ni_55.7_Ti_44.3_ alloyed component, but that the chemical composition of the build has a large heterogeneity. To solve this problem three different annealing heat treatments were designed, and the resulting porosity, microstructural homogeneity, and phase formation was investigated. The heat treatments were found to improve the alloy’s chemical and phase homogeneity, but the brittle NiTi_2_ phase was found to be stabilized by the 0.54 wt.% of oxygen present in all fabricated samples. As a consequence, a Ni_2_Ti_4_O phase was formed and was confirmed by transmission electron microscopy (TEM) observation. This study showed that pore formation in in situ alloyed NiTi can be controlled via heat treatment. Moreover, we have shown that the two-step heat treatment is a promising method to homogenise the chemical and phase composition of in situ alloyed NiTi powder fabricated by LPBF.

## 1. Introduction

Shape memory alloys (SMAs) have generated great interest in a diverse number of engineering applications. Nickel-titanium (NiTi), also referred to as Nitinol, is the most frequently used SMA that demonstrates a stable shape memory effect and superelastic behaviour, which allows for large recoverable strains of up to 8% [1,2]. Furthermore, it has good biocompatibility, damping characteristics, corrosion resistance, and a low Young’s Modulus (50–80 GPa) compared with other alloys commonly used in biomedical applications [3,4]. The specific mechanical properties of NiTi alloys render their machining a challenging task. One of the challenges of NiTi alloys is their extremely poor machinability resulting in rapid and uncontrollable tool wear and poor surface quality. This is due to the high ductility, superelasticity, low elastic modulus, and work hardening of the NiTi alloy the machined part is made of [5,6]. Demanding machining generates a high cost of the fabricated parts and limits the applicability and commercial usage of NiTi alloys. For this reason, an extremely important issue is to reduce material cost by eliminating machining, which would result in a lower cost of the fabricated elements. Thanks to additive manufacturing (AM) techniques that eliminate the need for machining, complex shapes of NiTi alloys have been produced in recent years [7,8,9]. Laser powder bed fusion (LPBF) is one of the powder-based AM methods allowing the fabrication of a wide variety of functional, complex three-dimensional shaped parts. In this technique the model is built directly from CAD data by melting successive layers of metal powder on top of the previous one, using thermal energy supplied by a focused and computer-controlled laser beam. It offers the opportunity to produce parts with complex geometries without resorting to cutting, pressing, grinding tools or fixtures. Since AM is a fast-growing industrial sector there is a strong need for new material development to satisfy the various engineering and medical application requirements. Despite its promise to overcome these challenges, the AM of NiTi has rarely been considered or explored for the production of NiTi devices. Current research into the AM of NiTi parts from pre-alloyed powders has been associated with difficulties concerning chemical homogeneity and chemical composition control caused by Ni evaporation during the melting process [10,11,12]. Since phase transition temperatures of NiTi alloys, which determine their unique properties, are highly sensitive to the alloy composition, any changes in the Ni/Ti ratio are undesirable [2,13]. One of the possible alternatives to solve the difficulties and reduce the costs of the production of a few different chemical compositions of NiTi powders (Ni/Ti ratio) is exploring the possibility of alloying Ni and Ti elemental powders in situ during the LPBF processes [14,15,16,17,18]. In situ alloying offers the flexibility to tailor the batch powder composition, by mixing the proper amount of pure metal powders [19,20]. The effect of in situ alloying during the LPBF process is the fabrication of the new object from a predesigned alloy composed of elemental powders [19,20]. For NiTi in situ fabrication, by knowing the amount of nickel evaporated during fabrication [2,9], the proper mixing ratio can be estimated in order to design the chemical composition of the final material [14]. Moreover, the use of nickel and titanium elemental powders is nearly three times cheaper than pre-alloyed NiTi powders currently available on the market. Thus, using elementally blended Ni and Ti powders would reduce the price of final objects.

**Figure 1:**
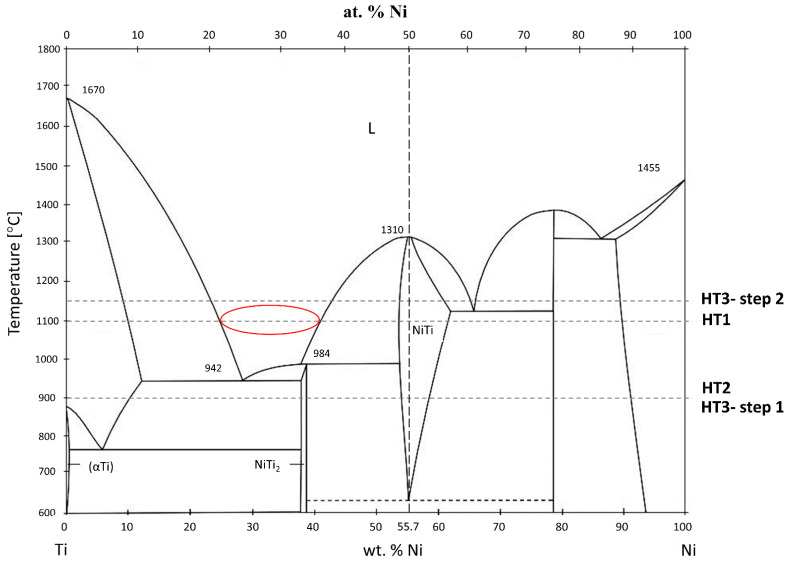
Phase diagram of the Ni-Ti system (based on [27]). The horizontal dashed lines represent the temperatures of the HT1–HT3 heat treatments; The vertical dashed line represents the composition of the initial Ni-Ti powder blend studied in this work; the red circle indicates the local concentrations for which there is a liquid phase at 1100 °C.

It has been reported that for NiTi AM-built objects using both elementally blended and pre-alloyed powders, a heat treatment is essential to improve the homogeneity, mechanical properties, and thermomechanical response [8,21]. As reported by Li et al. [21] and Lee et al. [22], the formation of the NiTi_2_ phase was observed in a NiTi alloy fabricated from pre-alloyed powder. In research work, where NiTi parts were AM-built from elementally blended powders, it was observed that the NiTi martensitic or austenitic, as well as NiTi_2_ phases, were present [8,14,15,16,17,18,23,24,25,26]. Moreover, the presence of secondary phases such as Ni_3_Ti and Ni_4_Ti_3_ were found in as-built specimens by Wang et al. [14] and Halani et al. [16], respectively. Hence, they investigated the influence of the heat treatment on the microstructure and phase composition of fabricated specimens. Based on the Ni-Ti phase diagram (Figure 1), the melting point of NiTi_2_ is 984°, thus, Wang et al. and Halani et al. subjected the as-built samples made from elementally blended NiTi powders to a solution heat treatment at 1000 °C for 6 h and 1050° for 10 h, respectively. Nevertheless, they found that unwanted secondary Ni_3_Ti and NiTi_2_ phases were formed instead of being eliminated in the material after heat treatment. 

In this study, a NiTi alloy was fabricated from elementally blended Ni and Ti powders using LPBF technique and was heat-treated to improve microstructure homogeneity. The influence of the different heat treatments on the microstructure and phase composition of the LPBF fabricated specimens was investigated. Moreover, the aim of this study was to design a heat treatment that homogenizes the microstructure and prevents porosity increase. Three heat treatments were proposed to achieve our research goals: (1) A one-step heat treatment at a temperature of 1100 °C; (2) a one-step heat treatment at a temperature of 900 °C; and (3) a two-step heat treatment, with the first step at a temperature of 900 °C and a second one at 1150 °C. Moreover, for the first time, the oxygen stabilization of the Ni_2_Ti phase that resulted in the formation of a thermodynamically stable Ni_2_Ti_4_O phase was revealed in the in situ alloyed NiTi. 

## 2. Materials and Methods

Powder of nickel (purity 99.9%, Eckart TLS, Bitterfeld-Wolfen, Germany) and titanium (grade 1 purity 99.7%, Eckart TLS, Bitterfeld-Wolfen, Germany) were mixed in a tumbling mixer for 2 h, without any additives, to achieve a homogenous mixture of the NiTi alloy having a composition of Ni_55.7_Ti_44.3_ which corresponds to pre-alloyed NiTi, used in our previous study [28]. For both powders, a particles size of <45 µm was used. A Realizer SLM 50 (Realizer GmbH, Borchen, Germany) was employed for the laser powder bed fusion (LPBF) fabrication of cylindrical samples with a diameter of 6 mm and a height of 10 mm. Argon gas was used as a protective gas and the oxygen level was kept below 0.3 vol.%. Samples were fabricated on a NiTi substrate which was maintained at 200 °C. An alternating scanning strategy including additional remelting after the first laser scanning was employed, thus each solidified layer was exposed to the laser beam two times. A laser power of 30 W and a scanning speed of 500 mm/s were used for first melting, while a slightly decreased laser power of 25 W and a scanning speed of 1000 mm/s were used for remelting. The layer thickness was set to 25 µm. The manufacturing procedure has been previously described in detail [10].

Annealing heat treatment (HT) was performed using an Elmor-1100 furnace (Falodlew, Gdańsk, Poland). This was carried out in a vacuum, using sealed quartz tubes using the parameters described in Table 1. The heating rate for each HT was fixed at 10 °C/min. After annealing, all samples were water quenched.

The samples were exposed to three different heat treatments denoted as HT1–HT3. The parameters of the HT1 heat treatment were chosen based on the literature [14,16,21] while HT2 and HT3 were chosen directly based on the NiTi phase diagram (Figure 1) to avoid liquid phase formation. The liquid metal has a bigger volume than its solid state, thus, the melt pool shrinking during solidification results in the formation of voids (pores). This phenomenon was observed for HT1 heat treatment and is discussed extensively in this article in the discussion section. Therefore, the aim behind the HT2 and HT3 heat treatments was to minimize liquid phase formation during annealing to prevent further void formation. The proposed HT1 and HT2 are single step annealing heat treatments at 1100 and 900 °C respectively, followed by immediate water quenching. The HT3 is a two-step heat treatment: firstly, samples were heated up to 900 °C and held for 24 h, then the temperature was increased to 1150 °C (based on the DTA results) and held for another 24 h. Annealing time for HT2 and HT3 was increased to 24 h based on the literature to extend the diffusion time of alloying elements according to Fick’s second law [29,30,31]. Samples were water quenched immediately after the second step of annealing. Water quenching was performed to retain the NiTi phase and prevent the formation of brittle intermetallic phases i.e., NiTi_2_, Ni_3_Ti, Ni_4_Ti_3_ [32,33,34].

After the heat treatment samples were mounted in epoxy and mechanically polished on a Saphire 550 grinding and polishing machine (ATM Qness GmbH, Mammelzen, Germany) using SiC grinding papers (from #600 to #2000), and subsequently polished using 0.1 µm alumina oxide suspensions. Light microscopy (LM) was performed on Axio Scope Microscope (Carl Zeiss Microscopy GmbH, Kelsterbach, Germany). Porosity was determined by the Archimedes’ principle. Each sample was weighed three times, non-consecutively, in air and water using an electronic balance with ±1 mg of accuracy. Scanning electron microscopy (SEM) observations using a backscattered electron (BSE) detector (SU-8000, Hitachi, Ibaraki, Japan) at 5 kV and energy dispersion spectroscopy (EDS) analysis at 10 kV were used to reveal the chemical composition. To determine the phases present, in the as-built NiTi alloy and after different heat treatments, X-ray diffraction (XRD) was performed at room temperature using a Bruker D8 Advance diffractometer (Bruker, Karlsruhe, Germany) with filtered Cu Kα (λ = 0.154056 nm) radiation. The XRD machine parameters were as follows: voltage 40 kV, current 40 mA, angular range 2Θ from 20° to 55°, step Δ2Θ 0.05° and dwell time 3 s. The XRD patterns were analysed using Bruker EVA software and a PDF-2 database (from the International Centre for Diffraction Data). A Labsys DTA/DSC (Setaram, Caluire-et-Cuire, France) differential thermal analysis (DTA) machine, with heating/cooling varying between room temperature to 1500 °C at a rate of 40°/min, was used to determine the NiTi_2_ transformation temperature. In order to identify phases present in the HT3 sample, a cross-sectional membrane (thin foil) of the selected region was prepared by a focused ion beam (FIB) system (NB5000, Hitachi, Ibaraki, Japan). Subsequently, their microstructure was studied using a transmission electron microscope (TEM) JEOL JEM 1200 (JEOL, Tokyo, Japan) operated at 120 kV. 

A TCH 600 Nitrogen/Oxygen/Hydrogen determinator (LECO, Benton Harbor, MI, USA) was used to determine the content of oxygen in all fabricated samples. The elements were converted to their oxidized form by utilizing the gas fusion method and the infrared absorption (IR) was used to measure combustion gases within a metallic sample. 

## 3. Results

### 3.1. Microscopic Observation and Phase Analysis

**Figure 2:**
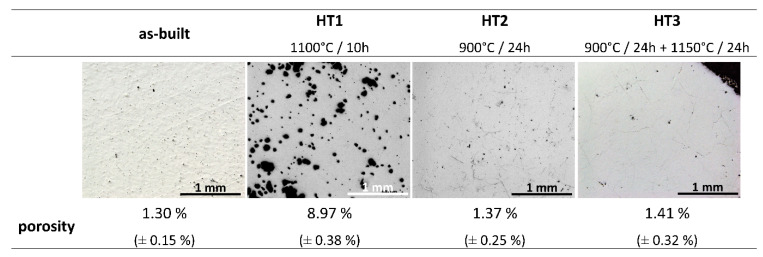
Light microscopic observation and porosity results of as-built sample; HT1 (1000 °C/10 h), HT2 (900 °C/24 h), and HT3 (900 °C/24 h + 1150 °C/24 h) heat-treated samples.

The as-built and heat-treated samples were subjected to light microscopic observations. Microstructures of all samples are shown in Figure 2. Pores are evident in each sample and show a spherical morphology. The porosity of the samples was determined by the Archimedes principle. For HT1 heat treatment, the phenomenon of the formation of the liquid phase, described in the discussion section, appeared in the sample. This caused the material porosity to increase remarkably. The porosity of the HT1 sample is almost seven times greater than that of the as-built sample. However, the porosity measurement results of the as-built sample, as well as HT2 and HT3 samples are similar. No significant influence of heat treatment on the porosity of the sample was noticed and the slight differences in porosity are within the measurement error (Figure 2). In addition, no increase in the number of cracks was observed in any of the heat-treated samples.

**Figure 3:**
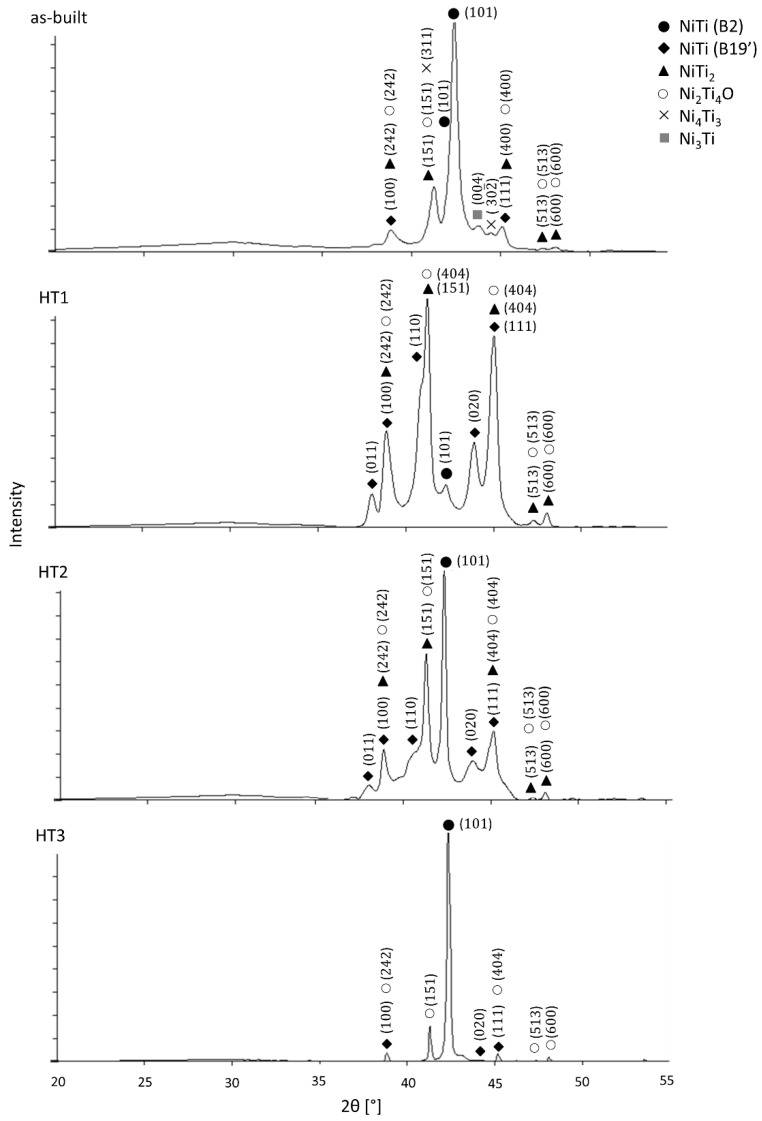
XRD diffractograms of as-built and HT1 (1000 °C/10 h), HT2 (900 °C/24 h), and HT3 (900 °C/24 h + 1150 °C/24 h) heat-treated samples.

The XRD results of the as-built and heat-treated samples are shown in Figure 3. The XRD patterns of the as-built sample showed the presence of multiple phases, including NiTi (B2) [35] and (B19′) [36], NiTi_2_ [37], Ni_2_Ti_4_O [37], Ni_3_Ti [38], and Ni_4_Ti_3_ [39]. A distinct change in the phases was observed in the heat-treated samples. After each heat treatment only NiTi (B19′), (B2), NiTi_2,_ and Ni_2_Ti_4_O phases were identified. Since NiTi (B19′), NiTi_2_ and Ni_2_Ti_4_O peaks overlap each other, it is not possible to distinguish them on XRD.

**Figure 4:**
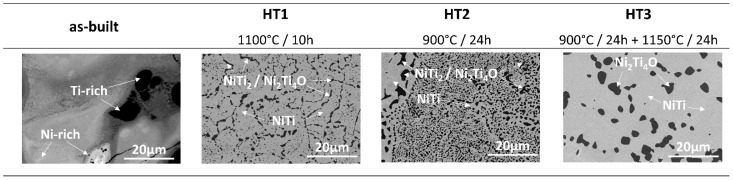
BSE SEM observation of the as-built sample; HT1 (1000 °C/10 h), HT2 (900 °C/24 h), and HT3 (900 °C/24 h + 1150 °C/24 h) heat-treated samples.

**Figure 5:**
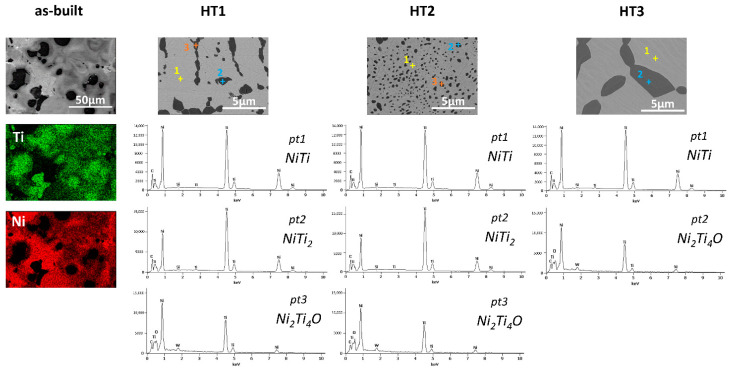
EDS mapping of the as-built sample indicates that the dark shaded areas are titanium-rich and bright shaded areas are nickel-rich; EDS point analysis revealed the presence of Ni, Ti, and O elements.

The phase composition homogeneity of the samples and the microstructures were captured by SEM BSE observations, as illustrated in Figure 4. The SEM BSE images of the as-built sample indicate a large heterogeneity of the chemical composition. EDS analysis (Figure 5) was performed to determine the Ni and Ti distribution in the as-built material. Distinct differences in contrast indicate the presence of titanium-rich (dark) and nickel-rich (bright) areas. In the case of heat-treated samples, only two different phases can be distinguished in the images, the dark-shaded titanium-rich phase, and the bright-shaded nickel-rich phase. EDS point analysis showed the presence of oxygen in some regions of a dark-shaded phase in samples HT1–HT3; however, the presence of oxygen was not detected in all regions of the phase in HT1 and HT2 samples. Therefore, the dark-shaded areas consist of NiTi_2_ and/or Ni_2_Ti_4_O phases. A more detailed analysis of the phase composition of the dark-shaded phase in each sample is discussed in paragraph 4. Based on the SEM BSE images, the percentage amount of the dark-shaded Ti-rich phases in each of the tested samples was calculated in the Micrometer software (Micrometer, Poland) using the method described by Wejrzanowski et al. [15,16] (Table 2). The greatest amount of dark-shaded phase is found in the HT2 sample (26 ± 4%), while the least amount was found in the HT3 sample (12.5 ± 0.5%). The amount of dark-shaded phase in the HT1 sample was 18 ± 2%. It was found that the dark-shaded phase in HT1 and HT2 samples is finer and has a more irregular shape than the phase in the HT3 sample, which is larger and has a nearly spherical shape. The presence of C, Si, and W elements detected by EDS (Figure 5) is a result of surface contamination that remains from materials used for surface polishing and preparation for TEM observations.

### 3.2. Differential Thermal Analysis 

**Figure 6:**
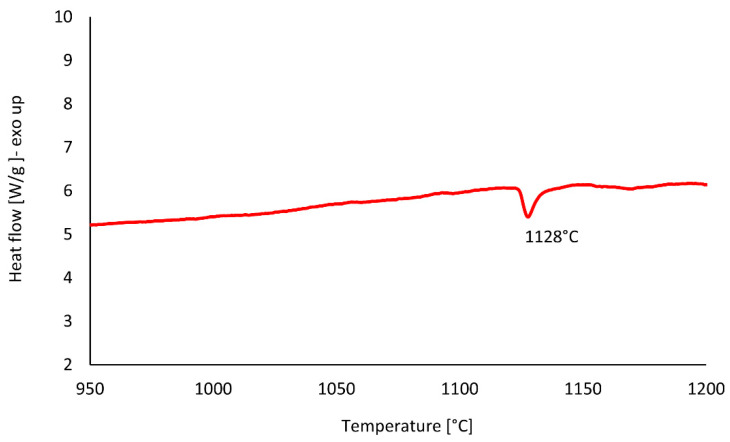
DTA trace of HT2 (900 °C/24 h) sample shows that NiTi_2_ melts at about 1128 °C and solidifies during cooling at about 1113 °C.

The Differential Thermal Analysis (DTA) test was performed on the HT2 sample to determine the NiTi_2_ phase transformation behaviour and indicate its melting temperature (Figure 6). The results indicate that the NiTi_2_ melts at about 1128 °C. 

### 3.3. Transmission Electron Microscopy 

**Figure 7:**
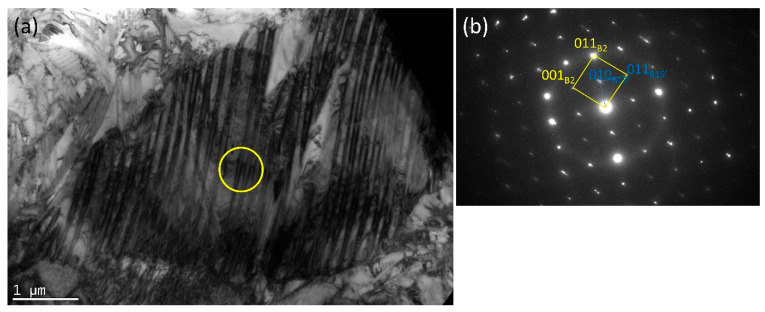
(**a**) A bright-field image of the NiTi matrix; (**b**) with a corresponding SAED pattern from (an area marked by a yellow circle).

**Figure 8:**
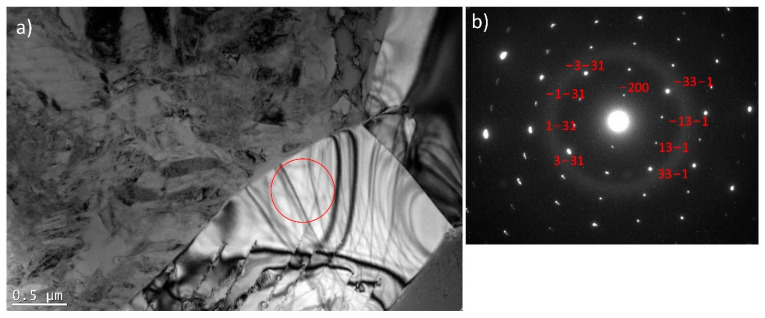
(**a**) TEM image of NiTi matrix with Ni_2_Ti_4_O particles; (**b**) SAED of Ni_2_Ti_4_O in the orientation [013] (an area marked by a red circle).

For a more detailed examination of the dark-shaded phase in the HT3 sample (Figure 4), (that was not removed after HT3, as expected) Transmission Electron Microscopy (TEM) was performed. Figure 7 shows a bright-field image of a NiTi matrix and a corresponding SAED pattern. The matrix consists of B2 cubic austenite and B19′ monoclinic martensite phases as presented by Dutkiewicz et al. [40]. The crystallographic relationship (001) B2 II (011) B19′and [100] B2II [100] B19′ results from the SAED pattern. Figure 8 shows particles present in the NiTi matrix with a corresponding selected area electron diffraction (SAED) from a single particle. One can expect to find NiTi_2_ or Ni_2_Ti_4_O (Figure 8) which are in the same crystal system and possess approximately the same lattice parameter. However, the differences between intensities of diffraction spots from {311} and {331} planes on the diffraction pattern enabled the identification of the Ni_2_Ti_4_O phase; a phase having XRD peaks overlapping NiTi_2_. Moreover, in the case of the NiTi_2_ phase, the intensity from the diffraction spot from {311} planes would be negligible contrary to the Ni_2_Ti_4_O phase.

### 3.4. Oxygen Content

The oxygen content in all samples was analysed to check whether the oxygen, which caused the formation of the Ni_2_Ti_4_O phase, could have dissolved in the sample during the LPBF fabrication or the heat treatment. The presence of 0.52 to 0.54 wt.% of the oxygen is observed in all samples (Table 3). There are no significant differences in the oxygen content between the as-built and heat-treated samples. Thereby, it can be assumed that oxygen was dissolved in the sample during the LPBF fabrication, and this stabilized the Ni_2_Ti_4_O phase.

## 4. Discussion

The Ni_55.7_Ti_44.3_ alloy was fabricated by laser powder bed fusion (LPBF) from elementally blended nickel and titanium powders and the high chemical and phase composition heterogeneity suggested that heat treatment is necessary to improve homogeneity. Therefore, three heat treatments were applied to solve this technical problem.

### 4.1. As-Built Sample

Microscopic observation of the as-built samples is presented in Figure 2. It shows that the sample is free of cracks or delamination and has a porosity of 1.30%. SEM observations in BSE mode showed the heterogeneity of the chemical composition of the sample (Figure 4). The Ti-rich and Ni-rich areas were revealed by EDS (Figure 5), meaning that components were not fully alloyed during laser melting. XRD phase analysis (Figure 3) indicated the presence of NiTi (B2) and NiTi (B19′), NiTi_2_, Ni_2_Ti_4_O, Ni_3_Ti, and Ni_4_Ti_3_ phases. Therefore, heat treatment of the manufactured alloy is necessary to homogenise its chemical and phase composition. 

### 4.2. First Heat Treatment—HT1

In the first heat treatment, HT1, the temperature and time were determined based on the literature. Wang et al. [14] employed LPBF to manufacture a NiTi alloy from elementally blended Ni and Ti powders and subjected the fabricated samples to a solution heat treatment at 1000 °C for 6 h and subsequently quenched in water. Halani and Shin [16] fabricated NiTi coupons using elementally blended powders by the flow-based direct energy deposition (DED) method and heat-treated them at 1050 °C for 10 h, with subsequent quenching in water. In the work presented by Li et al. [21] an additively manufactured NiTi alloy was heat-treated at temperatures that were determined based on DSC tests. It was concluded that NiTi_2_ in the alloy studied by Li et al. [21] has a melting temperature of 1010 °C. Thus, they performed heat treatment at a temperature close to the determined NiTi_2_ melting point (1010 °C). When analysing the NiTi phase diagram (Figure 1), it can be seen, that the widest range of NiTi phase presence (49–57 at.% of Ni) occurs at 1118 °C. The temperature of HT1 was determined based on the literature review and the NiTi phase diagram analysis. Therefore, HT1 was performed at 1100 °C for 10 h. Microscopic observation of the HT1 sample was shown in Figure 2 and the presence of large spherical pores was revealed. The porosity of the sample was 8.97%. EDS analysis showed the presence of the oxygen in some regions of a dark-shaded phase (Figure 5), however, the presence of the oxygen was not detected in all regions of the phase. SEM BSE observations (Figure 4), EDS analysis (Figure 5) and corresponding XRD analysis (Figure 3) determined the presence of NiTi (B2), NiTi (B19′), NiTi_2_ and Ni_2_Ti_4_O phases. The Ni_3_Ti and Ni_4_Ti_3_ phases on the other hand were eliminated. The amount of dark-shaded NiTi_2_ and Ni_2_Ti_4_O phases in HT1 sample was 18 ± 2%. Although the microstructure was significantly homogenized, the porosity of the sample increased almost seven times. 

Morris and Morris [41] examined different techniques for the sintering of Ni and Ti elemental powders. They found that when samples are sintered at high temperatures, where melting of a phase occurs, high porosity is generated in the material. Moreover, they noticed a large heterogeneity in the microstructure of sintered samples. They concluded that homogeneity of the material can be increased due to solid-state interfusion, occurring at temperatures below 900 °C, when no liquid phase appears in the material. In our study, the occurrence of Ti- and Ni-rich regions were presented in the as-built sample (Figure 4). According to the NiTi phase diagram (Figure 1), the liquid phase occurs at the temperature of 1100 °C in a wide range of compositions; especially 27 to 38 wt.% of Ni. The liquid phase of a material has a larger volume than its solid counterpart, therefore, during cooling, the material will shrink, resulting in voids. These voids induced the porosity that is observed in Figure 2. 

### 4.3. Second Heat Treatment—HT2

The second heat treatment, HT2, was aimed at homogenizing the microstructure and composition at a temperature that does not allow any phase to liquify since this could generate high porosity. Various conditions of material fabrication and, inter alia, the presence of internal stresses can influence the melting temperature (increase or decrease it) of materials [42]. To this effect, HT2 was performed at 900 °C, i.e., well below the lowest transition temperature of titanium-rich phases to the liquid state (942 °C). The HT2 time was extended to 24 h to increase the diffusion time according to Fick’s second law [29,30,31]. Microscopic observations and porosity measurement results (Figure 2) show that the porosity has not increased and is well below to that found in the HT1 sample. Thus, the reduction of the heat treatment temperature avoided pores formation caused by the melting of the Ti-rich phases. SEM BSE images (Figure 4) revealed the presence of two phases. The presence of oxygen was revealed by EDS in some areas of the dark-shaded phase in the HT2 sample (Figure 5). XRD analysis (Figure 3) confirmed that the samples consisted of B2, B19’, NiTi_2,_ and Ni_2_Ti_4_O phases. It has been observed that both HT2 and HT1 reduced the number of phases when compared to the as-built samples. The amount of dark-shaded phases in the HT2 sample was 26 ± 4% (Table 2). Due to the fact that HT1 was conducted at a temperature of 1100 °C which is close to the solubility of the NiTi_2_ phase in the fabricated material (1123 °C), more of it diffused into the surrounding phase than it was for HT2, which was carried out at a lower temperature (900 °C). Thus, the highest amount of dark-shaded NiTi_2_ and Ni_2_Ti_4_O phases remained in the HT2 sample.

Since NiTi_2_ is a brittle phase, it favours cracks formation and decreases the ductility [43] and it would be therefore beneficial to eliminate the presence of this phase. DTA analysis, shown in Figure 6, was performed to determine the melting temperature of the NiTi_2_ phase formed in the HT2 heat-treated sample. The melting temperature of the NiTi_2_ phase in the studied alloy was at 1128 °C. Based on this result, it was expected that the annealing heat treatment at a temperature slightly above the melting point of the NiTi_2_ phase will allow the diffusion of the elements and the elimination of the phase from the alloy. According to the Ni-Ti phase diagram (Figure 1), the liquid phase is present for a wide range of Ti concentrations (~75 to 60 wt.%) at 1100 °C. Direct annealing conducted above this temperature leads to pronounced, local melting of the material resulting in voids formation after the heat treatment routine. Initial annealing at temperatures where only solid phases exist activates diffusional homogenization and accelerates the formation of the ordered structures which are thermodynamically favoured over solid solutions due to low enthalpies of formation [44]. In such a scenario, the temperature of a second annealing step can be adjusted to eliminate the unwanted phase by heating the material slightly above the thermal stability of the selected, ordered structure. In this case, the amount of the liquid phase is minimal, and melted elements will diffuse to neighbouring phases and/or form other, intermetallic compounds stable under given thermodynamic conditions. To conclude, the HT2 heat treatment improved the homogenization of the chemical composition, however, the NiTi_2_ and Ni_2_Ti_4_O phases remained within the alloy. Therefore, the HT3 heat treatment has been designed and performed to eliminate the NiTi_2_ and Ni_2_Ti_4_O phases.

### 4.4. Third Heat Treatment—HT3

Based on the observations made and discussed above, the third heat treatment, HT3, was designed to eliminate from the alloy the NiTi_2_ phase through a two-step annealing treatment. Firstly, the samples were heated to 900 °C for 24 h to diffuse the elements and homogenise the chemical composition. Secondly, the samples were immediately heated to 1150 °C (no cooling between steps) and annealed for 24 h. After the second step was completed, samples were immediately water quenched. Since DTA analysis (Figure 6) showed that the NiTi_2_ phase melts at 1128 °C, it was expected that annealing just above the melting point, thus at 1150 °C, should allow the elements to diffuse into the NiTi phase and 1150 °C temperature was chosen for our study. The motivation behind the selection of the two annealing temperatures arises from the thermal stability of intermetallic compounds occurring in NiTi mixtures. It is expected that laser melting of commercially pure Ti and Ni elemental powders can lead to local Ti- or Ni-rich regions, which favour crystallisation of NiTi_2_, Ni_4_Ti_3,_ or Ni_3_Ti phases. The main goal behind the adopted annealing treatments is chemical homogenisation of the fabricated material, however, due to possible local inhomogeneity, the maximum temperature of heat treatment (1150 °C) was chosen slightly above the stability of NiTi_2_ (1128 °C) to minimize the risk of formation of a large amount of liquid phase inside the samples. Microscopic observations of the HT3 sample and porosity measurement results (Figure 2) showed that the porosity did not significantly increase compared with the as-built sample. SEM BSE observation (Figure 4) and the calculation of the dark-shaded phase volume fraction content (Table 2) revealed that when compared with the HT1 (18 ± 2%) and HT2 (26 ± 4%) samples there was a reduction of dark-shaded phase and its amount in the HT3 sample was 12.5 ± 0.5%. It should be noted that the phase was not completely eliminated. Moreover, EDS analysis revealed the presence of oxygen in all regions of the dark-shaded phase (Figure 5). Recent studies on NiTi alloys have suggested that the NiTi_2_ phase can be stabilized by oxygen. The presence of the Ni_2_Ti_4_O phase in NiTi alloy was reported by Kai et al. [45] and Frenzel et al. [13]. NiTi_2_ can absorb oxygen up to 14 at.% without changing its FCC crystal structure [46]. Oxygen can dissolve in the NiTi_2_ structure, and cause it to transform into a thermodynamically stable oxygen-rich Ni_2_Ti_4_O_x_ oxide phase [45]. Both NiTi_2_ and Ni_2_Ti_4_O are crystallographically very similar to each other, both possess FCC structure, thus, it is impossible to distinguish them apart using the XRD technique [47]. Hiroyuki et al. [46] have stated that the Ni_2_Ti_4_O phase in NiTi alloys cannot be eliminated even after two annealing heat treatments at 900 °C. In the HT3 sample NiTi (B2), NiTi (B19′), and NiTi_2_/Ni_2_Ti_4_O phases were identified by XRD (Figure 3). The XRD phase analysis did not indicate the presence of TiO_2_. However, it has been shown in our previous study that the thin layer of TiO_2_ was found on the material’s surface by X-ray photoelectron spectroscopy (XPS) [48]. Since the peaks corresponding to NiTi (B19′), NiTi_2_ and Ni_2_Ti_4_O overlap each other the XRD technique cannot be used alone to determine which phase is present in the studied alloy. To this effect, TEM was performed to confirm whether the analysed dark phase, shown in Figure 4, was NiTi_2_ or Ni_2_Ti_4_O. TEM analysis of the matrix revealed the presence of both B2 and B19′ phases (Figure 7). Furthermore, the differences between intensities of diffraction spots on the diffraction pattern enabled the identification of the dark phase as Ni_2_Ti_4_O (Figure 8). Based on this, the oxygen content in the chemical composition of the studied samples was determined and was found in all samples to be 0.52 to 0.54% (Table 3). Since the amount of oxygen in the as-built sample is comparable to all the heat-treated samples, it can be concluded that oxygen was introduced into the samples during the LPBF fabrication and not during heat treatment. The Realizer SLM50 machine used for samples fabrication does not provide a 100% pure oxygen-free atmosphere since the oxygen level in the building chamber during the fabrication process was about 0.3 vol.%. The oxygen residuals in the machine chamber caused oxygen pickup by the NiTi during the LPBF process, in a similar way to oxygen pickup by other materials reported in recent studies [28,49]. As was shown in this study, it is subsequently difficult to remove unwanted secondary phases from the material by applying heat treatments. The negative effect of oxygen residuals and other side effects of powder processing in an inert atmosphere can be eliminated during additive manufacturing processes by using a vacuum [50,51]. Vacuum LPBF processing, which is a relatively new technique for AM processes, could avoid the stabilization of the unwanted secondary phases, such as the NiTi_2_ phase. Therefore, further research should focus on the elimination of oxygen from the building chamber. Moreover, the issue of the as-built material heterogeneity can be eliminated during AM processes using machines that give the possibility to fabricate materials at elevated temperatures. For this reason, fabrication at elevated temperatures (above 500 °C) would be implemented to perform in situ heat treatment during the LPBF process. Consequently, the transformation temperatures of the fabricated materials would be further investigated using differential scanning calorimetry, as described by Martins et al. [52].

## 5. Conclusions

Laser powder bed fusion (LBPF), using a remelting scanning strategy and elementally blended Ni and Ti powders, was used to additively manufacture NiTi alloys. Since the as-built components possessed a heterogeneous chemical and phase composition, three different annealing heat treatments were designed and applied. The study led to the following main conclusions:

- The selected two-step HT3 heat treatment condition (900 °C/24 h + 1150 °C/24 h) allows for the significant homogenization of the chemical and phase composition of the LPBF in situ alloyed NiTi components;
- The Ti-rich phases in the as-built material melt during the chosen HT1 temperature (1100 °C) and upon solidification shrinkage occurs resulting in pore formation;
- Oxygen pickup during the LBPF manufacturing process promoted the formation of a thermodynamically stable, oxygen-rich Ni_2_Ti_4_O phase that is observed even after an annealing heat treatment;
- LPBF combined with post annealing is a promising way of fabricating NiTi alloys using elemental powder blends. Elimination of the oxygen pickup during the process and decrease of the possibility for formation of the oxides like Ni_2_Ti_4_O could be reached by undergoing the process in vacuum conditions, however further studies proving this hypothesis should be performed.

## Figures and Tables

**Table 1:** Parameters of the heat treatments of in situ alloyed NiTi fabricated by LPBF.

Name	Number of Steps	Temperature [°C]	Time [h]
**HT1**	1	1100	10
**HT2**	1	900	24
**HT3**	2	900 + 1150	24 + 24

**Table 2:** The volume fraction of NiTi_2_/Ni_2_Ti_4_O phases in HT1-HT3 samples.

	HT1	HT2	HT3
**NiTi_2_/Ni_2_Ti_4_O**	18	26	12.5
**volume fraction (%)**	±2	±4	±0.5

**Table 3:** Oxygen content in as-built and HT1-HT3 samples.

	As-Built	HT1	HT2	HT3
oxygen content (wt.%)	0.52 ±0.1	0.53 ±0.1	0.54 ±0.2	0.52 ±0.1

## Data Availability

Not applicable.
